# The relationship among hopelessness, suicide risk, body mass index and eating motivations in major depressive disorder comorbid with obesity: a case–control study

**DOI:** 10.1186/s12991-025-00580-y

**Published:** 2025-07-03

**Authors:** Fatma Gül Helvacı Çelik, Meltem Hazel Şimşek, Meltem Puşuroğlu, Ulaş Korkmaz

**Affiliations:** 1 Giresun University, Department of Psychiatry, Giresun, Turkey; 2 Recep Tayyip Erdoğan University, Department of Psychiatry, Rize, Turkey

**Keywords:** Major depressive disorder, Eating motivation, Hopelessness, Suicide risk

## Abstract

**Objective:**

Major Depressive Disorder (MDD) is a significant mental health problem, frequently comorbid with both physical and psychiatric disorders. The association between MDD and obesity is not fully understood. Eating motivations (EMs), which relate to why and how individuals choose to eat, may be associated with disorders like obesity and MDD. Hopelessness and suicidal ideation are common symptoms of MDD. This study aimed to evaluate the relationship between EMs, depression, body mass index (BMI), hopelessness, and suicidal ideation in normal-weight and obese MDD groups compared to healthy controls.

**Method:**

The study included 50 patients with MDD and normal weight (BMI 18.5–24.9), 50 patients with MDD and obesity (BMI > 30), and 50 healthy control participants (BMI 18.5–24.9). The majority of participants were women (74% in obese MDD, 70% in normal-weight MDD, 56% in the control group). The age of the groups was similar for the normal-weight MDD and control groups, but the obese MDD group was older (control: 32.72 ± 10.07, normal-weight MDD 33.42 ± 10.24, obese MDD 39.52 ± 10.67, p = 0.002). Regarding BMI, it was as follows: control: 21.61 ± 1.92, normal-weight MDD 23.54 ± 3.60, and obese MDD 35.30 ± 5.07. Sociodemographic data form, Beck Depression Inventory, Beck Hopelessness Inventory, Suicide Ideation Scale and Eating Motivation Questionnaire were administered.

**Results:**

No significant differences were found between the MDD groups in terms of Beck Depression Inventory (BDI), Beck Hopelessness Inventory (BHI), and Suicidal Ideation Scale (SIS) scores. Significant differences were observed among all groups in most subtypes of EMs. In the obese MDD group, habits, traditional eating, price, visual appeal, and affect regulation were correlated with suicide attempts. Only traditional eating remained associated with suicide attempts, where a one-unit increase in the traditional eating score explained a 0.724-unit increase in suicide attempts. Additionally, emotion regulation was a significant predictor of suicidal ideation in the obese MDD group, where a one-unit increase in emotion regulation explained a 0.885-unit increase in suicidal ideation.

**Conclusions:**

The differentiation between suicidal ideation and EMs in obese and normal-weight MDD groups is crucial. The observed differences in EMs among the three groups with similar sociocultural characteristics are noteworthy. Clinicians should assess eating motivations as part of suicide risk evaluations in patients with comorbid MDD and obesity. Longitudinal studies are needed to clarify causal relationships between these variables.

## Introduction

Major Depressive Disorder (MDD) is a significant mental health condition that continues to rank among the leading causes of disability worldwide, severely affecting functionality and quality of life (WHO, [45]). According to the World Health Organization [16](WHO) in 2023, approximately 3.8% of the global population experiences depression, affecting nearly 280 million individuals across all age groups (WHO, [46]; GHDx, [20]). MDD often co-occurs with numerous chronic and acute physical and psychiatric conditions, presenting complex challenges due to its multifactorial nature [33].

One important but often overlooked dimension of MDD involves alterations in eating behavior [42, 43]. Eating behavior encompasses not only the fulfillment of physiological needs but also emotional, social, and cultural motivations, collectively referred to as Eating Motivations (EMs) [21, 36]. Various biological, psychological, cultural, and environmental factors including appearance, taste, smell, and texture of food, can influence EMs [13, 21]. Emotional eating, a specific type of EM, is especially prominent in psychiatric conditions such as depression and anxiety [34]. Furthermore, appetite changes are recognized among the diagnostic criteria for MDD, highlighting the intricate relationship between depression and eating patterns (APA, [1]). Studies have shown that individuals with MDD often engage in emotional eating, further complicating symptomatology and prognosis [24].

Body Mass Index (BMI), a simple yet widely used measure calculated by dividing weight (kg) by height squared (m^2^), provides essential information about an individual’s weight category: underweight (≤ 18), normal weight (18.5–24.9), overweight (25–29.9), obesity (30–39.9), and morbid obesity (≥ 40) [44]. In psychiatric settings, BMI is crucial for monitoring conditions like eating disorders and tracking medication-induced weight changes [28, 47]. Importantly, emerging evidence suggests that depression, emotional eating, and obesity may form a mutually reinforcing cycle, in which mood disturbances affect eating behaviors, leading to weight gain, which in turn exacerbates depressive symptoms [24].

Hopelessness, defined as pervasive negative expectations about the future, is another critical factor closely linked to MDD [12]. Individuals experiencing hopelessness often feel powerless to solve problems or achieve goals, which intensifies depressive symptoms and predicts worse clinical outcomes [6, 22]. Hopelessness not only reflects depression severity but also significantly elevates the risk of suicidal ideation and behaviors [9, 37]. Hopelessness has been associated not only with depression but also with emotional eating behaviors [38]. Hopelessness may exacerbate the severity of depression, triggering emotional eating, which may contribute to the development of obesity. Alternatively, a reciprocal relationship may occur, where obesity leads to depression and hopelessness.

Suicide, a major global public health issue, claims approximately one million lives annually and is the second leading cause of death among individuals aged 10 to 34 (WHO, 2017–2018; [40]). According to WHO data from 2021, around 703,000 deaths were attributed to suicide (WHO, [46]). MDD is a major contributor to suicide risk, with an estimated 15% of individuals with depression attempting or completing suicide during depressive episodes [10],WHO 2017–2018; [32].

Given these complex and interrelated factors, the aim of this study is to assess the relationships between EMs, depression severity, BMI, hopelessness, and suicidal ideation in individuals diagnosed with MDD. Both normal-weight and obese MDD patients will be compared to healthy controls. We hypothesize that eating motivations (EMs) will differ significantly among the three groups (normal-weight MDD, obese MDD, and healthy controls), with the MDD groups showing stronger associations between certain eating motivations (e.g., emotional eating, traditional eating) and depression severity, as compared to the healthy control group. Another hypothesize is variations in EMs will correlate with differences in hopelessness and suicidal ideation in MDD groups. Furthermore, it is proposed that within the MDD population, different types of EMs may differentially influence depression severity, hopelessness, and suicidal ideation. By simultaneously evaluating EMs and BMI alongside psychological factors in individuals with MDD, this study aims to contribute to a more integrated understanding of the disorder and inform novel therapeutic approaches.

## Materials and methods

The study included individuals who presented to the psychiatry clinic and were diagnosed with MDD, as well as a healthy control group with similar sociodemographic characteristics and convenience sampling method used. The participants were categorized into three groups: normal-weight depression, obese depression, and healthy control. The patient group was classified based on BMI into normal weight (BMI 18.5–24.9) and obese (BMI > 29.9) categories. Individuals with underweight (BMI ≤ 18) and overweight (BMI 25–29.9) status were excluded from the study. The study included a total of 150 participants. All participants in the three groups completed the following assessments: a sociodemographic data form (SDF), Beck Depression Inventory (BDI), Beck Hopelessness Inventory (BHI), Suicidal Ideation Scale (SIS), and Eating Motivation Questionnaire (EMQ). Additionally, the height and weight of participants were measured to calculate BMI and categorize them into their respective groups.

The patient group included individuals diagnosed with MDD, with no comorbid mental illnesses, no history of psychiatric treatment in the past year, and no chronic internal medical conditions. Participants were required to have sufficient mental capacity and education to complete the scales. The healthy control group consisted of individuals with normal BMI values and no history of chronic mental illness or any internal medical conditions (e.g., endocrinological, cardiac, or decompensated physical illnesses) that could affect the data. Control participants were randomly selected from the general population. Individuals who were unable to provide informed consent due to cognitive impairment or other factors impacting their decision-making ability were excluded from the study.

All participants, both in the patient and control groups, provided written and verbal consent to participate in the study. A specialized psychiatrist conducted psychiatric assessments using a structured clinical interview form (SCID-5) based on DSM-5 criteria to exclude comorbid psychiatric conditions and ensure accurate diagnoses. The SCID-5 is a widely used diagnostic tool that provides a comprehensive evaluation based on the Diagnostic and Statistical Manual of Mental Disorders, Fifth Edition (DSM-5) criteria [15]. The study received ethical approval from the Recep Tayyip Erdoğan University Non-Interventional Ethics Committee (E-40465587-050.01.04-630, dated 23.02.2023).

### Sociodemographic data form

This form, created by the researchers, evaluates variables such as gender, age, education, income level, height-weight, employment status, psychiatric history and suicidal history.

### Beck depression inventory (BDI)

This is a 21-item Likert-type self-report scale used to assess the severity of depression [4]. The scale includes items related to mood, behavior, and physical symptoms of depression. A higher score indicates more severe depressive symptoms. For instance, one example item is: *“I feel sad or blue most of the time”*. Scores range from 0 (not at all) to 3 (severely), and the total score is indicative of the level of depression. The Turkish version of the BDI has been validated for both reliability and validity [18]. Cronbach’s alpha for the BDI is typically reported as 0.87, indicating good internal consistency.

### Beck hopelessness inventory (BHI)

This is a 20-item Likert-type scale designed to assess an individual’s level of pessimism about the future [5]. It measures negative expectations about the future, including beliefs about personal goals, future events, and general outlook. A high total score on this scale indicates a high level of hopelessness. An example item is: *“I have very little confidence in the future”*. The Turkish version of the BHI has been validated for reliability and validity [14]. Cronbach’s alpha for the BHI is 0.89, which indicates a high degree of internal consistency.

### Suicidal ideation scale (SIS)

This is a 17-item self-report scale that evaluates the presence and intensity of suicidal thoughts and behaviors [26]. The scale covers aspects such as suicidal planning, thoughts, attempts, and past suicide history. The score is interpreted in relation to clinical data, and higher scores suggest an increased likelihood of suicidal ideation. An example item is: *“Have you had thoughts of ending your life in the past week?”*. The Turkish validity and reliability of the scale have been established [3]. Cronbach’s alpha for the SIS is 0.91, indicating excellent internal consistency.

### Eating motivation questionnaire (EMQ)

This is a self-report tool used to assess the motivations behind eating behaviors [21]. It includes various sub-dimensions, such as healthy eating and emotional eating, to better understand the different factors driving food choices. One example item is: *“I eat when I am feeling stressed or anxious”*. The Turkish validity and reliability of the EMQ have been established [23]. Cronbach’s alpha for the EMQ is 0.85, indicating good internal consistency.

## Statistical analysis

In the power analysis, the total number of samples to be taken in a difference analysis to be conducted with 3 groups with an alpha value of 0.05, an effect size of 0.5 and 80% power was calculated as 150 people with a 10% margin of error and a 95% confidence interval. Since no study with specific mean and standard deviation values for our study could be found in the literature, the effect size was taken as 0.5 (medium effect size). The data were evaluated statistically with the “SPSS Statistics 21” program. Frequency, percentage values, median (interquartile range), mean and standard deviation values were determined as descriptive statistics. Skewnes and kurtosis evaluations were performed to determine normal distributions. As suggested by Tabachnick and Fidell, if the Skewness and Kurtosis values were between − 1.5 and + 1.5, the data was considered to comply with normal distribution [11]. For comparisons, Student's T-Test and ANOVA were used for numerical variables that followed a normal distribution, and Mann Whitney U test and Kruskal Wallis-H test were used for numerical variables that did not follow a normal distribution. Comparisons between categorical variables were made with the chi-square test. Comparative correlation analyzes (Pearson for those with normal distribution, Spearman for those with non-normal distribution) were performed to determine the variables affecting the numerical data. If the correlation values are between 0–0.3, they are interpreted as very low level, between 0.3–0.5, they are interpreted as low level, between 0.5–0.7, they are interpreted as medium level, between 0.7–0.9, they are interpreted as high level, and between 0.9–1.0, they are interpreted as very high-level correlation [41]. p < 0.05 was considered statistically significant [17].

## Results

The study included 50 obese (BMI > 30) and 50 normal weight (BMI < 25) MDD patients, as well as 50 healthy individuals with normal weight. The majority of the patient group and healthy controls were women (74% in obese MDD, 70% in normal weight MDD, 56% in control group). The ages of the groups were similar in normal weight MDD and control group, but obese MDD group is higher (control 32.72 ± 10.07, normal weight MDD 33.42 ± 10.24 and obese MDD 39.52 ± 10.67, p = 0.002). Regarding BMI, it was determined as follows: control 21.61 ± 1.92, normal weight MDD 23.54 ± 3.60, and obese MDD 35.30 ± 5.07. In all groups, the majority of individuals were married, had received 9 years or more of education, had an average income, lived in nuclear families and had a job (Table 1).

**Table 1:** Comparison of sociodemographic data between groups

	Obese MDD (n:50)	Normal weight MDD (n:50)	Control group (n:50)	X^2^	p
Gender				4.020	0.134
Women	37 (%74)	35 (%70)	28 (%56)		
Men	13 (%26)	15 (%30)	22 (%44)		
Education level				14.744	0.005
0–8 years	12 (%24)	11 (%22)	5 (%10)		
9–12 years	22 (%44)	25 (%50)	14 (%28)		
> 12 years	14 (%28)	14 (%28)	31 (%62)		
Marital status				16.551	< 0.001
Single	11 (%22)	20 (%40)	31 (%62)		
Married	39 (%78)	30 (%60)	19 (%38)		
Employment status				6.424	0.04
Employed	22 (%44)	21 (%42)	11 (%22)		
Unemployed	28 (%56)	29 (%58)	39 (%78)		
Income level				11.310	0.073
None	11 (%22)	17 (%34)	6 (%12)		
Min. wage	5 (%10)	3 (%6)	3 (%6)		
2–3Xmin. wage	17 (%349	20 (%40)	29 (%48)		
> 3Xmin. wage	17 (%34)	10 (%20)	12 (%24)		

MDD Major Depressive Disorder; X^2^:Chi-square; p: significance value

In obese MDD patients, the presence of a psychiatric history, suicidal thoughts, suicide attempt, and a family history of suicide attempts were found to be higher compared to normal weight MDD patients. The data related to the psychiatric history and suicide evaluations of the groups are listed in Table 2.

**Table 2:** Psychiatric History and Suicide Variables of the Groups

	Obese MDD(n:50)	Normal weight MDD (n:50)	Control group(n:50)	X^2^	p
Psychiatric history				13.801	< 0.001
No	22 (%44)	29 (%58)	40 (%80)		
Yes	28 (%56)	21 (%42)	10 (%10)		
Psychiatric hospitalization				1.895	0.362
No	46 (%92)	49 (%98)	50 (%100)		
Yes	4 (%8)	1 (%2)	0 (%0)		
Suicidal thoughts				42.00	< 0.001
No	20 (%40)	30 (%60)	50 (%100)		
Yes	30 (%60)	20 (%40)	0 (%0)		
Suicide attempt				18.267	< 0.001
No	34 (%68)	38 (%76)	50 (%100)		
Yes	16 (%32)	12 (%24)	0 (%0)		
Family history of suicide attempt				16.031	< 0.001
No	36 (%72)	47 (%94)	48 (%96)		
Yes	14 (%28)	3 (%6)	2 (%4)		

MDD Major Depressive Disorder; X^2^: Chi-square; p: significance value

There were no significant differences between the obese and normal weight MDD groups in terms of BDI, BHI, and SIS scores. However, significant differences were observed among all groups in most subtypes of EMQ (Tables 3 and 4).

**Table 3:** Comparison of BDI, BHI, and SIS in Obese, and Normal weight MDD Groups

	Healty control (n:50)	Normal weight MDD (n:50)	Obese MDD(n:50)	F	Effect size	p
	Mean ± St. deviation	Mean ± St. deviation	Mean ± St. deviation			
BDI	*	35.26 ± 9.82	35.92 ± 9.27	0.119	0.001	0.730
BHI	*	11.74 ± 3.97	11.12 ± 4.27	0.563	0.006	0.455
SIS	*	7.8 ± 3.31	7.98 ± 4.00	0.060	0.001	0.807

MDD Major Depressive Disorder; BMI Body Mass Index; BDI: Beck Depression Inventory; BHI: Beck Hopelessness Inventory; SIS: Suicide Ideation Inventory; p: significance value; St. Deviation: standart deviation; n: number

* This symbol indicates that the healthy control group was not included in the analysis

**Table 4:** Comparison of EMQ subscales scores in healthy control, obese, and normal weight MDD groups

	Healty control (*n*:50)	Normal weight MDD (*n*:50)	Obese MDD (*n*:50)	F	Effect size	p
	Mean ± St. Deviation	Mean ± St. Deviation	Mean ± St. Deviation			
Liking	3.84 ± 1.50	4.36 ± 1.25	5.25 ± 1.20	14.553	0.165	** < 0.001**
Habits	3.59 ± 1.33	4.09 ± 1.50	5.10 ± 1.07	17.025	0.188	** < 0.001**
Need & Hunger	3.52 ± 1.22	3.74 ±.1.36	4.36 ± 1.15	6.028	0.076	**0.003**
Health	3.89 ± 1.22	2.99 ± 1.49	2.55 ± 1.23	13.461	0.155	** < 0.001**
Convenience	3.29 ± 1.17	3.86 ± 1.50	4.37 ± 1.38	7.873	0.097	**0.001**
Pleasure	3.23 ± 1.56	3.15 ± 1.49	4.44 ± 1.25	12.444	0.145	** < 0.001**
Traditional Eating	3.53 ± 1.19	3.37 ± 1.40	4.09 ± 1.45	3.939	0.051	**0.022**
Natural Concerns	3.61 ± 1.34	3.01 ± 1.71	2.69 ± 1.35	4.895	0.062	**0.009**
Sociability	2.30 ± 0.92	2.15 ± 1.23	2.88 ± 1.41	5.115	0.065	**0.007**
Price	2.30 ± 1.06	2.63 ± 1.45	2.98 ± 1.53	3.092	0.040	**0.048**
Visual Appeal	2.76 ± 0.99	2.95 ± 1.28	4.06 ± 1.57	14.414	0.164	** < 0.001**
Weight Control	3.27 ± 1.41	2.60 ± 1.46	2.12 ± 1.08	9.364	0.113	** < 0.001**
Emotion Regulation	2.20 ± 1.14	3.22 ± 1.67	4.81 ± 1.67	37.350	0.337	** < 0.001**
Social Norms	2.35 ± 0.77	2.50 ± 1.20	3.62 ± 1.22	20.377	0.217	** < 0.001**
Social Image	1.80 ± 0.72	1.81 ± 1.02	2.41 ± 1.30	5.647	0.071	**0.004**

MDD Major Depressive Disorder; BMI Body Mass Index; BDI: Beck Depression Inventory; BHI: Beck Hopelessness Inventory; SIS: Suicide Ideation Inventory; EMQ: Eating Motivation Questionnaire; p: significance value; St. Deviation: standart deviation; n: number

The bold font in Table 4 indicates statistically significant results (p .05)

Habits, traditional eating, price, visual appeal, affect regulation, BMI correlated with suicide attempt in the obese MDD group. In the normal weight MDD group, only habits, pleasure, traditional eating and social norms correlated with BDI scores (Table 5).

**Table 5:** Significant variables with correlation in the obese and normal weight MDD groups

Obese MDD (n:50)					Normal weight MDD (n:50)	
EMQ	Suicide attempt	SIS	BDI	BHI	EMQ	BDI
Habits	r = 0.356 p = 0.049	r = 0.427 p = 0.002	–	–	Habits	r = 0.327 p = 0.021
Health	–	r = −0.28 p = 0.047	–	–	Pleasure	r = 0.282 p = 0.047
Traditional Eating	r = 0.518 p = 0.003	–	–	–	Traditional Eating	r = 0.311 p = 0.028
Price	r = 0.420 p = 0.019	–	–	–	Social Norms	r = 0.315 p = 0.026
Visual Appeal	r = 0.384 p = 0.033	–	–	–		
Emotion Regulation	r = 0.462 p = 0.009	r = 0.45 p = 0.001	r = 0.382 p = 0.006	–		
Social Norms	–	–	–	r = −0.304 p = 0.032		
BMI	r = 0.396 p = 0.028	–	–	–		

MDD Major Depressive Disorder: BMI Body Mass Index: BDI: Beck Depression Inventory: BHI: Beck Hopelessness Inventory: SIS: Suicide Ideation Inventory: EMQ: Eating Motivation Questionnaire; p: significance value; r: correlation coefficient

Habits, traditional eating, price, visual appeal, emotional regulation, and BMI variables, which were found to be associated with suicide attempts in our study, were entered into the multiple linear regression analysis performed to predict suicide attempt in the obese group. In the model, the normality assumption was met and multicollinearity was not detected (VIF < 5). According to the model, a one-unit increase in the traditional eating score explains a 0.724-unit increase in suicide attempts (95% CI = 0.170–1.278). Other variables did not have a significant effect. The model explains 28% of the variance (Table 6).

**Table 6:** Multiple regression analysis results for suicide attempts in the obese MDD group

Independent variable	F	Adjusted R^2^	p	B	95% CI
	2.927	0.278	0.028		
Constant				− 1.309	− 4.985–2.367
Habits				− 0.517	− 1.294–0.260
Traditional Eating				0.724	0.170–1.278
Price				0.137	− 0.158–0.431
Visual Appeal				− 0.199	− 0.574–0.175
Emotion Regulation				0.344	− 0.142–0.831
BMI				0.015	− 0.090–0.121

MDD Major Depressive Disorder: BMI Body Mass Index; p: significance value; CI: confidence interval

Habits, health, and affect regulation variables, which were found to be associated with SIS in our study, were entered into the multiple linear regression analysis performed to predict SIS scores in the obese MDD group. In the model, the normality assumption was met and multicollinearity was not detected (VIF < 5). According to the model, a one-unit increase in the emotion regulation score explains a 0.885-unit increase in the SIS score (95% CI = 0.248–1.521). Other variables did not have a significant effect. The model explains 20% of the variance (Table 7).

**Table 7:** Multiple regression analysis results for SIS scores in the obese MDD group

Independent variable	F	Adjusted R^2^	p	B	95% CI
	5.018	0.197	0.004		
Constant				1.011	− 4.899–6.922
Habits				0.778	− 0.201–1.757
Health				− 0.493	− 1.341–0.354
Emotion Regulation				0.885	0.248–1.521

MDD Major Depressive Disorder; p: significance value; CI: confidence interval

In the obese MDD group, it was found that as age increased, the motivation subgroups of pleasure and liking decreased (p = 0.009, r = − 0.366; p = 0.005, r = − 0.390 respectively), and as depression increased, emotion regulation increased (p = 0.004, r = 0.404).

## Discussion

This study examined the relationship between eating motivation, depression, BMI, hopelessness, and suicidal ideation in individuals diagnosed with MDD. To date, there is no study that evaluates all of these variables together in the context of MDD in the literature. Furthermore, studies that evaluate both normal-weight and obese MDD patients and compare them with healthy populations are limited. In this regard, our study holds particular significance.

While the relationship between depression and obesity has been extensively explored, it remains an area of complexity. Some studies suggest that depression leads to weight gain and obesity, while others argue that obesity may cause depression. Most research indicates bidirectional causality between the two [25, 31]. Additionally, there are findings pointing to an inverse relationship between obesity and depression [27]. However, studies assessing the relationship between BMI and depression remain relatively scarce. For example, a study conducted in Turkey found that the severity of depression increased as BMI increased, suggesting a potential relationship between higher BMI and more severe depressive symptoms [29]. Another study found that weight loss was associated with a decrease in depression scores in individuals who were obese but did not have clinical depression [7]. There is also literature addressing the relationship between obesity and suicide. A systematic review found an inverse relationship between obesity and suicide-related deaths, but a positive relationship between obesity, suicidal thoughts, and planning [2]. A more recent meta-analysis involving 45 low and middle income countries indicated that overweight and obesity were significantly associated with an increased risk of suicidal thoughts and suicide attempts, particularly in youth [48]. In our study, however, we found no significant differences in depression severity, hopelessness, or suicidal ideation between obese and normal-weight MDD patients. The lack of significant differences may be due to the small sample size or specific characteristics of the sample.

The examination of EMs across different cultures and countries is an underexplored area of research. While some studies focus on EMs in specific situations, few explore the psychiatric aspects of EMs, particularly within psychiatric settings [35, 39]. In Turkey, research on EMs has largely been limited to the assessment of questionnaire validity and reliability, without focusing on the psychiatric implications of EMs [3, 8]. In our study, significant differences in most subtypes of EMs were found between the three groups. These differences were observed even among groups from the same country and region, which share similar sociocultural patterns. This highlights the importance of evaluating EMs in diverse populations and patient groups, particularly in the context of psychiatric treatment and healthy eating strategies. Such evaluations can significantly contribute to a deeper understanding of EM variations.

A notable finding in this study is the correlation between subgroups of EMs (e.g., habits, traditional eating, price, visual appeal, and affect regulation) and suicide attempts in the obese MDD group. While the relationship between BMI and emotional regulation is expected, there is a need for studies with larger sample sizes and diverse cultural contexts, as no previous studies in the literature have explored this relationship in depth. When performing a more detailed analysis, only traditional eating maintained its significant relationship with suicide attempts. Specifically, a one-unit increase in the traditional eating score was associated with a 0.724-unit increase in suicide attempts. This suggests that a higher tendency toward traditional eating might reflect a lack of openness to innovation or adaptation, potentially linked to weaker coping mechanisms in the face of depression or life difficulties, which could lead to an increased risk of suicide attempts.

In the normal-weight MDD group, no significant correlations were found between suicide attempts and EM subgroups. This suggests that differences in EMs, depression, and suicide may exist between obese and normal-weight MDD patients.

When evaluating suicidal ideation, we found that only an increase in emotion regulation eating motivation predicted suicidal ideation in the obese MDD group. Specifically, a one-unit increase in emotion regulation eating motivation was associated with a 0.885-unit increase in the Suicidal Ideation Scale score. Emotional eating has become an increasingly prominent area of research in psychiatric literature, with many studies suggesting that emotional eating may act as a mediator in the relationship between depression and obesity. One study discovered a positive association between depression and emotional eating, both of which were predictive of an increase in BMI [24]. Furthermore, depression history and its severity are linked to increased levels of emotional and uncontrolled eating [30]. In our study, the finding that emotional eating predicted suicidal ideation only in the obese MDD group supports this existing body of literature.

Another important finding in the obese MDD group is that as age increases, the subgroups of pleasure and liking decrease, while emotion regulation increases. These findings suggest that changes in EMs may be more pronounced with age among individuals with obese MDD. The variations observed in the obese MDD group highlight the potential impact of age-related factors on eating behaviors and motivations in this specific population.

Additionally, in the obese MDD group, as the severity of depression increases, eating problems may also escalate, underscoring the need for interventions that address both emotional well-being and eating behaviors in this population. This emphasizes the importance of a holistic approach to treatment, one that considers the complex interplay between depression, emotional eating, and obesity.

Our study revealed significant differences in eating motivations among the three groups. Notably, EMs such as traditional eating and emotional regulation were strongly associated with suicidal ideation in the obese MDD group. The effect size for the relationship between traditional eating and suicidal ideation was moderate. Similarly, the effect size for emotional regulation's impact on suicidal ideation in the obese MDD group was substantial. These findings suggest that certain eating motivations may serve as important predictors of suicidal ideation in obese MDD patients, highlighting the need for interventions targeting these behaviors in clinical settings. The findings also emphasize the role of emotional eating and traditional eating behaviors in the context of depression and obesity.

## Limitations of the study

Despite the important insights gained from this study, several limitations should be acknowledged. The cross-sectional nature of the study limits our ability to draw conclusions about causality. Longitudinal studies are necessary to clarify whether certain eating motivations directly lead to worsened depressive symptoms, hopelessness, or suicidal ideation, or whether these behaviors are simply associated with these outcomes. While a control group was included, individuals with low body weight, overweight, and morbid obesity were excluded from the study. This limits the generalizability of the findings, as the sample did not represent the full spectrum of BMI categories. Future research should consider including individuals from all BMI categories to provide a more comprehensive understanding of the relationship between depression, eating motivations, and BMI. Some sociodemographic differences between the groups may present a limitation. These differences could affect the interpretation of the findings, and future studies should take into account more detailed sociodemographic factors to control for potential confounding variables. Although a power analysis was conducted, the sample size in this study is relatively small. A larger sample size would provide greater statistical power and increase the reliability of the findings. The use of self-report scales can be considered a limitation, as they rely on individuals'subjective perceptions, which may be influenced by social desirability bias or inaccurate self-assessment. The fact that the study was conducted at a single center limits the generalizability of the results. Future research should aim to include multiple centers to ensure that the findings are applicable to a wider population. The control group in this study consisted only of individuals with normal weight, which may limit the ability to draw comparisons with individuals in other BMI categories. Including individuals with varying BMI values in future control groups could provide a more nuanced understanding of the relationship between eating motivations, BMI, and depression. The lack of significant differences in depression severity and hopelessness between the obese and normal-weight MDD groups warrants further investigation. Potential moderating factors such as comorbid conditions, treatment histories, or genetic predispositions should be explored in future studies to help refine therapeutic strategies. Future research should consider examining the impact of cultural and contextual factors on eating motivations and mental health. Understanding how these factors influence eating behaviors and depression could lead to more personalized and effective treatment strategies.

## Strengths of the study

In the literature, there is no study that evaluates all of these variables in major depressive disorder. Assessments of eating motivation are generally limited. Moreover, comparisons between obese and non-obese MDD groups are very limited. Our study is valuable for all of these reasons and can serve as a model for future research. The finding that eating motivations are so different among three groups with similar sociocultural characteristics is noteworthy. Another important result is the differentiation of comparisons related to suicide in the obese and non-obese MDD groups.

## Conclusion

The findings of this study have important practical implications for therapeutic strategies and public health policies. Given the observed correlation between eating motivations, depression, and suicidal ideation, therapeutic interventions that address both mental health and eating behaviors could be particularly effective for patients with comorbid MDD and obesity. For example, incorporating eating behavior interventions such as mindful eating, nutritional counseling, and emotional regulation strategies into standard depression treatments could potentially reduce both depressive symptoms and the risk of suicide. These interventions could help patients better cope with emotional distress without resorting to unhealthy eating behaviors, thus improving their overall well-being.

From a public health perspective, these findings underline the need for a more integrated approach to treating depression in individuals with obesity. Policy makers should consider developing programs that promote mental health support alongside obesity prevention and treatment initiatives. In particular, community-based interventions that address both psychological and physical health aspects could have a profound impact on reducing the burden of MDD and obesity, especially in high-risk populations.

## Data Availability

Data available on request from the authors.
